# Supplementary material for: Low Childhood Nature Exposure is Associated with Worse Mental Health in Adulthood
Source: Int J Environ Res Public Health. 2019 May 22;16(10):1809. doi: 10.3390/ijerph16101809 (PMC6572245; doi:10.3390/ijerph16101809)
Supplement: Supplementary file 1 [file ijerph-16-01809-s001.pdf]

International Journal of
Environmental Research and Public Health

MDPI

Supplementary Material

Low Childhood Nature Exposure is Associated with Worse Mental Health in Adulthood

Myriam Preuß, Mark Nieuwenhuijsen, Sandra Marquez, Marta Cirach, Payam Dadvand, Margarita Triguero-Mas, Christopher Gidlow, Regina Grazuleviciene, Hanneke Kruize and Wilma Zijlema

Table S1. Mixed effects regression of the potential mediators (perception of amount of NOE, use of NOE, satisfaction with NOE, importance of NOE, residential surrounding greenness in 300m) on mental health (0-100 scale; higher is better mental health). Pooled and city-specific analyses.

		Crude model[a]	Adjusted model[b]
	n	β-coefficient (95% CI)	β-coefficient (95% CI)
Perception of amount of NOE			
Pooled	3578	1.44 (0.31, 2.57)*	0.65 (-0.44, 1.75)
Barcelona	982	2.50 (0.29, 4.72)*	2.21 (0.13, 4.29)*
Doetinchem	848	1.54 (-0.31, 3.38)	0.89 (-0.90, 2.68)
Kaunas	892	2.56 (0.23, 4.88)*	1.69 (-0.57, 3.95)
Stoke-on-Trent	856	-0.32 (-2.50, 1.87)	-1.61 (-3.79, 0.58)
Use of NOE			
Pooled	3483	2.44 (1.38, 3.50)***	2.10 (1.07, 3.13)***
Barcelona	970	2.49 (0.46, 4.52)*	2.78 (0.83, 4.74)**
Doetinchem	840	3.06 (1.00, 5.11)**	2.17 (0.18, 4.15)*
Kaunas	817	0.91 (-1.41, 3.23)	1.16 (-1.14, 3.46)
Stoke-on-Trent	856	3.96 (1.80, 6.12)***	2.58 (0.39, 4.77)*
Satisfaction with NOE			
Pooled	3574	2.87 (1.79, 3.94)***	2.12 (1.07, 3.17)***
Barcelona	977	4.89 (2.86, 6.91)***	4.38 (2.42, 6.34)***
Doetinchem	848	3.44 (1.44, 5.44)**	2.86 (0.93, 4.78)**
Kaunas	892	2.85 (0.58, 5.11)*	1.73 (-0.49, 3.95)
Stoke-on-Trent	857	0.00 (-2.43, 2.44)	-0.48 (-2.88, 1.92)
Importance of NOE			
Pooled	3578	2.18 (1.14, 3.21)***	2.17 (1.15, 3.19)***

Barcelona	981	2.69 (0.71, 4.66)**	2.61 (0.70, 4.52)**
Doetinchem	847	0.05 (-1.89, 1.99)	0.59 (-1.30, 2.47)
Kaunas	892	2.18 (-0.16, 4.52)	1.13 (-1.20, 3.48)
Stoke-on-Trent	858	3.97 (1.82, 6.12)***	3.73 (1.63, 5.84)**
Residential surrounding greenness in 300m			
Pooled	3583	1.58 (0.12, 3.04)*	1.11 (-0.27, 2.49)
Barcelona	983	-0.25 (-3.21, 2.71)	-1.35 (-3.96, 1.27)
Doetinchem	848	0.55 (-1.35, 2.45)	-0.20 (-2.03, 1.62)
Kaunas	892	-0.90 (-3.15, 1.36)	-0.48 (-2.71, 1.76)
Stoke-on-Trent	860	1.73 (-0.96, 4.43)	0.85 (-1.73, 3.43)

* $p \leq 0.05$; ** $p < 0.01$; *** $p < 0.001$

[a] Unadjusted model with random intercepts

[b] Adjusted for age, gender, origin, education, perceived financial situation, SES of the neighborhood, household composition, smoking status

CI, confidence interval; n, number of participants; NOE, natural outdoor environments

Table S2. Mixed effects regression of the mediators (perception of amount of NOE, use of NOE, satisfaction with NOE, importance of NOE, residential surrounding greenness in 300m) on vitality (0-100 scale; higher is better vitality). Pooled and city-specific analyses.

		Crude model[a]	Adjusted model[b]
	n	β-coefficient (95% CI)	β-coefficient (95% CI)
Perception of amount of NOE			
Pooled	3578	2.50 (1.21, 3.79)***	1.76 (0.49, 3.02)**
Barcelona	980	1.74 (-0.79, 4.26)	1.60 (-0.83, 4.02)
Doetinchem	850	2.57 (0.27, 4.88)*	2.11 (-0.13, 4.36)
Kaunas	892	2.30 (-0.03, 4.63)	1.57 (-0.69, 3.83)
Stoke-on-Trent	856	2.67 (-0.04, 5.39)	1.70 (-0.99, 4.39)

Use of NOE			
Pooled	3483	3.19 (1.96, 4.41)***	2.92 (1.73, 4.11)***
Barcelona	968	2.46 (0.14, 4.79)*	2.73 (0.47, 4.99)*
Doetinchem	842	5.45 (2.88, 8.02)***	4.21 (1.71, 6.71)**
Kaunas	817	2.13 (-0.21, 4.46)	2.63 (0.33, 4.92)*
Stoke-on-Trent	856	5.28 (2.59, 7.97)***	4.02 (1.30, 6.73)**
Satisfaction with NOE			
Pooled	3574	4.35 (3.12, 5.58)***	3.47 (2.26, 4.67)***
Barcelona	975	4.82 (2.49, 7.14)***	4.26 (1.99, 6.53)***
Doetinchem	850	2.06 (-0.46, 4.57)	1.35 (-1.09, 3.79)
Kaunas	892	3.59 (1.33, 5.86)**	2.47 (0.25, 4.68)*
Stoke-on-Trent	857	1.47 (-1.57, 4.52)	1.52 (-1.44, 4.49)
Importance of NOE			
Pooled	3578	2.53 (1.33, 3.72)***	2.74 (1.57, 3.92)***
Barcelona	979	2.26 (0.01, 4.52)*	2.79 (0.59, 4.99)*
Doetinchem	849	0.82 (-1.62, 3.26)	1.62 (-0.76, 3.99)
Kaunas	892	0.89 (-1.46, 3.24)	0.01 (-2.33, 2.34)
Stoke-on-Trent	858	5.93 (3.24, 8.62)***	5.39 (2.78, 8.00)***
Residential surrounding greenness in 300m			
Pooled	3583	0.92 (-0.74, 2.57)	0.50 (-1.08, 2.08)
Barcelona	981	1.07 (-2.23, 4.37)	-0.29 (-3.46, 2.88)
Doetinchem	850	1.23 (-1.08, 3.55)	0.35 (-1.91, 2.61)
Kaunas	892	-0.26 (-2.54, 2.03)	0.28 (-1.96, 2.52)

Stoke-on-Trent	860	2.03 (-1.11, 5.18)	1.10 (-1.84, 4.04)

* $p \leq 0.05$; ** $p < 0.01$; *** $p < 0.001$

[a] Unadjusted model with random intercepts

[b] Adjusted for age, gender, origin, education, perceived financial situation, SES of the neighborhood, household composition, smoking status

CI, confidence interval; n, number of participants; NOE, natural outdoor environments

Table S3. Mixed effects regression of childhood NOE exposure (low levels vs. high levels) on mental health and vitality (0-100 scales; higher is better mental health/vitality) with additional adjustment for residential surrounding greenness in 100m and 500m (separately). Pooled and city-specific analyses.

	n[a]	Additional adjustment for residential surrounding greenness in 100m β-coefficient (95% confidence interval)	Additional adjustment for residential surrounding greenness in 500m β-coefficient (95% confidence interval)
Mental health			
Pooled	3583	-4.12 (-5.51, -2.73)***	-4.11 (-5.50, -2.73)***
Barcelona	983	-3.90 (-6.30, -1.49)**	-3.90 (-6.31, -1.49)**
Doetinchem	848	-0.82 (-4.29, 2.65)	-0.79 (-4.26, 2.68)
Kaunas	892	-3.69 (-7.12, -0.26)*	-3.61 (-7.03, -0.18)*
Stoke-on-Trent	860	-5.48 (-7.88, -3.07)***	-5.48 (-7.88, -3.07)***
Vitality			
Pooled	3583	-1.27 (-2.88, 0.34)	-1.27 (-2.88, 0.34)
Barcelona	981	-1.96 (-4.73, 0.81)	-1.96 (-4.73, 0.82)
Doetinchem	850	-0.40 (-4.79, 3.99)	-0.38 (-4.77, 4.01)
Kaunas	892	-3.74 (-7.16, -0.32)*	-3.78 (-7.19, -0.37)*

Stoke-on-Trent	860	0.17 (-2.84, 3.18)	0.22 (-2.79, 3.23)

* $p \leq 0.05$; ** $p < 0.01$; *** $p < 0.001$

[a] n was the same for the residential surrounding greenness in a radius of 100m and 500m

CI, confidence interval; n, number of participants

Table S4. Mixed effects regression of childhood NOE exposure (low levels vs. high levels) on mental health and vitality (0-100 scales; higher is better mental health/vitality) with additional adjustments for the perception of amount of NOE, use of NOE, and satisfaction with NOE. Pooled and city-specific analyses. With a different cut off for childhood NOE exposure (sensitivity analysis). [a]

	Additional adjustment for perception of amount of NOE<br>β-coefficient (95% CI), n	Additional adjustment for use of NOE<br>β-coefficient (95% CI), n	Additional adjustment for satisfaction with NOE<br>β-coefficient (95% CI), n
Mental Health			
Pooled	-3.43 (-4.47, -2.38)***, 3578	-3.58 (-4.63, -2.53)***, 3483	-3.36 (-4.40, -2.32)***, 3574
Barcelona	-1.72 (-3.73, 0.29), 982	-1.75 (-3.77, 0.27), 970	-1.53 (-3.51, 0.45), 977
Doetinchem	-2.63 (-4.72, -0.53)*, 848	-2.60 (-4.69, -0.50)*, 840	-2.31 (-4.39, -0.23)*, 848
Kaunas	-2.44 (-4.65, -0.23)*, 892	-2.97 (-5.26, -0.68)*, 817	-2.36 (-4.57, -0.14)*, 892
Stoke-on-Trent	-6.12 (-8.21, -4.03)***, 856	-6.20 (-8.25, -4.14)***, 856	-6.28 (-8.35, -4.21)***, 857
Vitality			
Pooled	-0.90 (-2.11, 0.31), 3578	-1.72 (-4.37, 0.94), 3483	-0.80 (-2.01, 0.41), 3574
Barcelona	0.49 (-1.82, 2.80), 980	0.37 (-1.95, 2.70), 968	0.59 (-1.70, 2.89), 975
Doetinchem	-1.79 (-4.44, 0.85), 850	-0.52 (-4.90, 3.86), 842	-1.50 (-4.14, 1.14), 850

Kaunas	-2.84 (-5.05, -0.64)*, 892	-2.97 (-5.27, -0.66)*, 817	-2.76 (-4.97, -0.55)*, 892
Stoke-on-Trent	0.33 (-2.29, 2.95), 856	0.57 (-2.01-3.15), 856	0.17 (-2.41, 2.76), 857

* $p \leq 0.05$; ** $p < 0.01$; *** $p < 0.001$

[a] Note: Cut offs used for childhood NOE exposure were: "never", "sometimes", "regularly" as low levels of childhood NOE exposure (n=1369; 38.19%) and "often", "very often" as high levels of childhood NOE exposure (n=2216; 61.81%; reference group).

CI, confidence interval; n, number of participants; NOE, natural outdoor environments.

Table S5. Mixed effects regression of childhood NOE exposure on mental health and vitality (0-100 scales; higher is better mental health/vitality) with additional adjustments for importance of NOE, residential surrounding greenness in 300m, and all mediators combined. Pooled and city-specific analyses. With a different cut off for childhood NOE exposure (sensitivity analysis). [a]

	Additional adjustment for importance of NOE β-coefficient (95% CI), n	Additional adjustment for residential surrounding greenness in 300m β-coefficient (95% CI), n	Additional adjustment for all mediators combined[a] β-coefficient (95% CI), n
Mental health			
Pooled	-3.34 (-4.38, -2.29)***, 3578	-3.39 (-4.44, -2.35)***, 3583	-3.54 (-4.60, -2.49)***, 3464
Barcelona	-1.84 (-3.85, 0.18), 981	-1.88 (-3.88, 0.11), 983	-1.47 (-3.50, 0.56), 961
Doetinchem	-2.61 (-4.71, -0.51)*, 847	-2.69 (-4.79, -0.60)*, 848	-2.29 (-4.38, -0.20)*, 839
Kaunas	-2.30 (-4.52, -0.08)*, 892	-2.32 (-4.54, -0.10)*, 892	-3.05 (-5.33, -0.76)**, 817
Stoke-on-Trent	-6.10 (-8.15, -4.04)***, 858	-6.13 (-8.19, -4.06)***, 860	-6.33 (-8.41, -4.24)***, 847
Vitality			

Pooled	-0.70 (-1.91, 0.51), 3578	-0.84 (-2.05, 0.37), 3483	-0.84 (-2.07, 0.38), 3464
Barcelona	0.57 (-1.75, 2.88), 979	0.35 (-1.95, 2.64), 981	0.82 (-1.53, 3.17), 959
Doetinchem	-1.75 (-4.41, 0.91), 849	-1.86 (-4.52, 0.79), 850	-1.39 (-4.05, 1.27), 841
Kaunas	-2.70 (-4.91, -0.49)*, 892	-2.71 (-4.93, -0.50)*, 892	-3.00 (-5.30, -0.70)*, 817
Stoke-on-Trent	0.72 (-1.85, 3.29), 858	0.68 (-1.92, 3.29), 860	0.07 (-2.51, 2.65), 847

* $p \leq 0.05$; ** $p < 0.01$; *** $p < 0.001$

[a] "All mediators" includes: perception of amount of NOE, use of NOE, satisfaction with NOE, importance of NOE, NDIV in 300m

[a] Note: Cut offs used for childhood NOE exposure were: "never", "sometimes", "regularly" as low levels of childhood NOE exposure (n=1369; 38.19%) and "often", "very often" as high levels of childhood NOE exposure (n=2216; 61.81%; reference group).

CI, confidence interval; n, number of participants; NOE, natural outdoor environments.

© 2019 by the authors. Submitted for possible open access publication under the terms and conditions of the Creative Commons Attribution (CC BY) license (http://creativecommons.org/licenses/by/4.0/).
